# Ginseng polysaccharides ameliorate DSS-induced inflammatory bowel disease by regulating gut microbiota in dogs

**DOI:** 10.3389/fvets.2025.1708594

**Published:** 2026-01-08

**Authors:** Liuwei Xie, Xiao Li, Aipeng Mao, Zhiqiang Han, Xiuli Zhang, Xin Liu, Qing Liu, Weigang Zhao, Chao Xu

**Affiliations:** 1 College of Police Dog Technology, Criminal Investigation Police University of China, Shenyang, China; 2 College of Animal Science and Technology, Jilin Agricultural University, Changchun, China; 3 College of Veterinary Medicine, Jilin Agricultural University, Changchun, China; 4 Animal Nutrition Institute of Sichuan Agricultural University, Chengdu, China; 5 College of Veterinary Medicine, Jilin University, Changchun, China; 6 Institute of Special Animal and Plant Sciences, Chinese Academy of Agricultural Sciences, Changchun, China

**Keywords:** ginseng polysaccharide, gut microbiota, inflammatory bowel disease, serum parameters, short-chain fatty acids

## Abstract

Inflammatory bowel disease (IBD) is a common chronic gastrointestinal disorder in dogs that seriously affects health and quality of life. This study evaluated the effects of ginseng polysaccharides on dextran sodium sulfate (DSS)-induced IBD in dogs, with emphasis on clinical symptoms, serum parameters, and gut microbiota. Our findings revealed that treatment with ginseng polysaccharides alleviated clinical symptoms, improved colonic histopathology, and partially restored serum biochemical changes, including a significant reduction in C-reactive protein. Microbiota analysis showed increased alpha diversity and recovery of community composition, with enrichment of beneficial genera such as *Bacteroides*, *Megamonas*, and *Fusobacterium*, and reduction of *Campylobacter*. Functional prediction indicated that ginseng polysaccharides reversed DSS-associated suppression of carbohydrate metabolism pathways. These findings suggest that ginseng polysaccharides mitigate DSS-induced IBD in dogs by modulating inflammation and gut microbiota composition, supporting their potential as a natural therapeutic candidate for canine IBD.

## 1 Introduction

Dogs were among the first animals domesticated by humans and have long supported people in a wide range of working roles (1). Over time, sustained cooperation has created a strong human-dog bond, shifting the primary role of dogs from labor to companionship. As modern lifestyles have changed, dogs have become increasingly integrated into the family, and greater attention is now placed on their health and well-being (2).

Inflammatory bowel disease (IBD) is one of the most common chronic gastrointestinal disorders in dogs, characterized by persistent diarrhea, vomiting, and weight loss, which can severely impact overall health (3). Although the exact pathogenesis remains unclear, current research points to genetic, immune, and environmental factors (4, 5). Dogs with IBD exhibit a range of intestinal histopathological lesions, including mucosal fibrosis, crypt dilation, villus stunting, and inflammatory cell infiltration (6). Prolonged systemic inflammation can also lead to various extraintestinal complications, such as anemia, arthritis, and kidney or liver diseases (7). Furthermore, IBD patients often exhibit an imbalance in gut microbiota; however, a causal link between dysbiosis and disease development has not yet been established (8–10). Due to its protracted course, IBD often causes significant suffering in dogs. Despite extensive research, an effective treatment for IBD has yet to be developed. Currently, anti-inflammatory drugs, particularly glucocorticoids, are commonly used for IBD treatment (11). However, a substantial number of dogs treated with corticosteroids develop resistance and experience severe side effects (11). Therefore, there is an urgent need for new and more effective therapeutic options for canine IBD.

Ginseng, an herbaceous perennial belonging to the family *Araliaceae* and genus *Panax*, is known as the “king of herbs” (12). Ginseng polysaccharide, one of its primary active ingredients, is widely used in medicine and healthcare for its diverse bioactivities, including antitumor, antioxidant, gut-microbiota-regulating, and immunomodulatory effects (13, 14). A recent study found that ginseng polysaccharides and their acidic fraction ameliorated symptoms of DSS-induced colitis in rats by reducing the expression of inflammatory cytokines, maintaining intestinal barrier integrity, and modulating the diversity and composition of the gut microbiota (15). Another study reported similar results, showing that ginseng polysaccharides mitigated the development of DSS-induced ulcerative colitis in mice by inhibiting inflammatory cytokine expression and influencing the tryptophan metabolism of the intestinal microbiota (16). These findings highlight the promising potential of ginseng polysaccharides in the prevention or treatment of colitis. Ginseng and its active components also exert broader beneficial effects on the gut microbiota (17). For instance, ginseng polysaccharides restored gut homeostasis by enriching *Lactobacillus* and *Muribaculum intestinale* while suppressing the pro-inflammatory genus *Alistipes*, thereby demonstrating antitumor activity against *Aspergillus sydowii*-induced lung adenocarcinoma (18). Studies in dogs have shown similar microbiota-related benefits. Red ginseng dietary fiber appears to have prebiotic properties, increasing gut microbiota diversity in a dose-dependent manner, raising the abundance of short-chain-fatty-acid-producing bacteria, and reducing the abundance of potential pathogens, ultimately supporting gut health (19). Another study found that supplementation with black ginseng and silkworm improved serum total cholesterol and triglyceride levels in diet-induced overweight dogs and altered the *β*-diversity of the gut microbiota (20).

Dextran sodium sulfate (DSS) is a compound with anticoagulant properties that damages colonic epithelial cells, disrupts intestinal barrier integrity, exposes luminal antigens to mucosal and submucosal immune cells, and induces a marked inflammatory and immune response (21). DSS-induced enteritis models are among the most widely used models of IBD because they closely resemble the pathological features of ulcerative colitis and offer a high modeling success rate and a short experimental period (22, 23). Prednisone is a commonly used glucocorticoid. After being converted to its active form, prednisolone, in the liver, it is routinely administered for the treatment of moderate to severe active IBD (24). Its therapeutic effects involve inhibition of nuclear factor-κB activity, reduction of pro-inflammatory cytokines such as tumor necrosis factor-*α*, interleukin-1β, and interferon-*γ*, and stimulation of intestinal epithelial cell proliferation (25, 26). Prednisone produces rapid and observable improvements, including reduced inflammatory cell infiltration, crypt restoration, and alleviation of clinical signs such as diarrhea, hematochezia, and body weight loss, usually within a few days. Owing to these rapid effects, prednisone/prednisolone is often used as a positive control in DSS-induced IBD studies and provides a suitable benchmark for assessing anti-inflammatory efficacy in short-term DSS experiments (27, 28). In this study, a DSS-induced canine IBD model was constructed to investigate the ameliorative effects of ginseng polysaccharides on colitis symptoms, blood parameters, and gut microbiota. This study aims to provide a reference for the potential application of ginseng polysaccharides in the treatment of canine IBD.

## 2 Materials and methods

### 2.1 Materials

The ginseng polysaccharide (Cat# MXE20220410) was acquired from Shaanxi Mixianer Biotechnology Co., Ltd. (Xi’an, Shaanxi, China). The total polysaccharide content (98.6%) was determined using the phenol-sulfuric acid colorimetric method by the Institute of Quality Standards and Testing Technology Research Center of the Institute of Special Animal and Plant Sciences, Chinese Academy of Agricultural Sciences (Changchun, Jilin, China). DSS (MW 36,000–50,000 Da; Cat# 0216011025) and azoxymethane (Cat# A5486) were purchased from MP Biomedicals (Solon, OH, United States) and Sigma-Aldrich Corp. (St. Louis, MO, United States), respectively.

### 2.2 Ethics statement

All procedures involving dogs were conducted in strict accordance with the Guidelines for the Care and Use of Laboratory Animals, as required by the Chinese Legislation on Laboratory Animals and Chinese Academy of Agricultural Sciences. Efforts were made to ensure animal welfare, relieve pain, and reduce the number of dogs used. The study protocol was reviewed and approved by the Ethics Committee of the Laboratory Animal Administration of the Institute of Special Animal and Plant Sciences, Chinese Academy of Agricultural Sciences (Approval No. ISAPSAEC-2021-60D).

### 2.3 Animals and experimental design

A total of 12 healthy, 3-month-old male dogs with an average body weight of 1.90 ± 0.13 kg (mean ± SD) were selected for this study. The animals were obtained from a breeding facility in Changchun, China. All dogs were mixed breed and came from three litters with distinct genetic backgrounds. Before the experiment began, the dogs underwent a five-day acclimation period. Their health status was monitored daily to confirm that all individuals were suitable for inclusion. All dogs were then randomly assigned to four groups based on their genetic background and body weight to minimize the influence of genetic variation on experimental outcomes. Each group consisted of three dogs: the control group (CON, water only), the model group (MOD, 2.5% DSS only), the ginseng polysaccharide group (GP, 2.5% DSS + 0.35 g/kg ginseng polysaccharides), and the prednisone group (P, 2.5% DSS + 2 mg/kg prednisone). The dosage of ginseng polysaccharides was determined based on the conversion from the effective dose used in mice (29). All animals were housed in a controlled environment at 22 ± 2 °C with 40–60% relative humidity and a 16 h light and 8 h dark cycle. The dogs were fed ad libitum twice day (at 8:00 and 18:00). The composition and nutritional levels of the experimental diets are presented in Table 1.

**Table 1:** Composition and nutrient levels of the basal diet.

Items	Content (%)
Ingredients	
Extruded corn	39.00
Soybean meal	20.00
Corn germ meal	12.00
Distillers dried grains with solubles	10.00
Fish meal	4.00
Soybean oil	4.00
Extruded soybean meal	2.00
Meat meal	2.00
Hemoglobin power	2.00
Calcium hydrogen phosphate	1.50
Calcium carbonate	1.50
Premix (air-dry basis)^a^	0.80
Lysine	0.75
Methionine	0.25
Salt	0.20
Total	100.00
Nutrient levels^b^	
Gross energy/(MJ/kg)	18.39
Crude protein	24.37
Ether extract	7.70
Ash	7.72
Calcium	1.22
Phosphorus	1.34

^a^The premix provided the following per kilogram of the diet: Vitamin A 2,500 IU; Vitamin D3 400 IU; Vitamin E 24 IU; Vitamin B1 5 mg; Vitamin B2 3.6 mg; Vitamin K3 0.8 mg; Vitamin B6 3 mg; Vitamin B12 0.009 mg; Vitamin C 25 mg; biotin 0.004 mg; folic acid 0.2 mg; nicotinic 10 mg; choline chloride 360 mg; calcium pantothenate 6.8 mg; Fe 38.4 mg; Cu 7.2 mg; Mn 19.2 mg; Zn 31.2 mg; I 0.576 mg; Se 0.12 mg; Co 0.096 mg.

^b^The nutrient levels were measured values.

**Figure 1:**
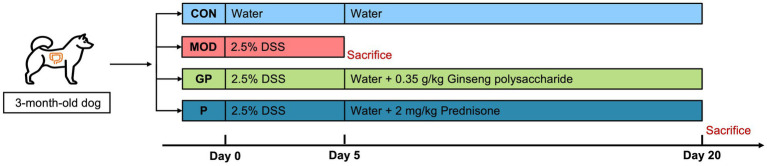
Flow of the trial. Control group (CON), model group (MOD), ginseng polysaccharide group (GP), and prednisone group (P). For the first 5 days, the CON group received drinking water, while the other groups received 2.5% DSS. For the next 15 days, the CON group continued to receive drinking water, while the GP and P groups were treated with 0.35 g/kg ginseng polysaccharides and 2 mg/kg prednisone, respectively.

The experimental flow is illustrated in Figure 1. During the first 5 days, the CON group received clean drinking water, while the MOD, GP, and P groups received 2.5% DSS in their drinking water to induce a canine IBD model (30). After this period, the dogs in the MOD group were anesthetized with propofol and subsequently euthanized by intravenous injection of a saturated potassium chloride solution. Colonic mucosa tissues and serum samples were then collected and stored at −80 °C. Colonic tissue samples were preserved in 4% paraformaldehyde, and colon length was measured. For the following 15 days, the CON group continued to receive clean drinking water, while the GP and P groups were treated with 0.35 g/kg ginseng polysaccharides and 2 mg/kg prednisone, respectively. At the end of the experiment, colonic mucosa, serum, and colonic tissue samples were collected from the CON, GP, and P groups using the same procedures.

During the trial, body weight was recorded daily, and the severity of colitis was evaluated using the Canine Inflammatory Bowel Disease Activity Index (CIBDAI) (31). Based on CIBDAI scores, clinical symptoms were categorized into four levels: clinically insignificant (0–3), mild (4–5), moderate (6–8), and severe (≥9). The experiment was conducted under controlled room conditions, including regulated temperature, humidity, and a 12-h light–dark cycle. All dogs were housed in individual cages, fed twice daily (at 7:00 and 16:00), and provided with ad libitum access to water throughout the study. Every effort was made to minimize the number of animals used and to reduce their suffering as much as possible throughout the study.

### 2.4 Hematoxylin and eosin (HE) staining

The specific experimental steps for HE staining were carried out as described in a previous study (30).

### 2.5 Blood parameters determination

Serum parameters, including total protein (TP), albumin (ALB), globulin (GLOB), aspartate aminotransferase (AST), alanine aminotransferase (ALT), alkaline phosphatase (ALP), lactate dehydrogenase (LDH), urea (UREA), calcium (Ca), phosphorus (P), and C-reactive protein (CRP), were measured using an SMT-120 V automatic biochemical analyzer (Chengdu Seamaty Technology Co., Ltd., Chengdu, Sichuan, China).

### 2.6 Intestinal microbial analysis

The colonic mucosa samples were sent to Novogene Co., Ltd. (Beijing, China) for intestinal microbiota analysis. Total genomic DNA was extracted using the SDS method. The V3-V4 region of the 16S rRNA gene was amplified using primers 341F (5’-CCTAYGGGRBGCASCAG-3′) and 806R (5’-GGACTACNNGGGTATCTAAT-3′). Sequencing was performed on the Illumina NovaSeq 6,000 platform. Species annotation was conducted using QIIME2 software and the Silva Database (v.138.1). To assess the richness and diversity of the intestinal microbial communities, alpha diversity metrics, including the Shannon, Pielou_e, and Simpson indices, were calculated in QIIME2. Differences in community composition between groups were evaluated using principal component analysis (PCA), performed with the ade4 and ggplot2 packages in R (version 3.5.3). To identify significantly different taxa at the phylum and genus levels, LEfSe (version 1.0) was applied using an LDA score threshold of 4.0 to determine the corresponding biomarkers. Functional prediction of microbial communities was conducted using PICRUSt to annotate and compare potential functional pathways across groups.

### 2.7 Short-chain fatty acids (SCFAs) analysis

The levels of SCFAs, including acetic, propionic, and butyric acids, were determined using a gas chromatography system (Agilent Technologies Inc., Santa Clara, CA, United States), following procedures to described in our previous work (32). Briefly, about 0.2 g of wet digesta was combined with 6 mL of ultrapure water and kept at 4 °C for 6 h. The mixture was then centrifuged at 5000 × g for 10 min at 4 °C to obtain the supernatant. A 25% (w/v) metaphosphoric acid solution was added to the extract at a 1:5 volume ratio. Afterward, the mixture was centrifuged again at 12,000 × *g* for 15 min at 4 °C, and the resulting supernatant was filtered through a 0.22 μm membrane before SCFA quantification. SCFAs were separated on an Agilent HP5 silica capillary column (30 m × 0.32 mm × 0.32 μm). The temperature was programmed to start at 60 °C, increase at 10 °C/min to 170 °C, and then rise at 8 °C/min to a final temperature of 212 °C. Nitrogen of high purity served as the carrier gas. The injector temperature was set at 250 °C, and the detector temperature at 270 °C.

### 2.8 Statistical analysis

GraphPad Prism 10 (GraphPad Software, Inc., San Diego, CA, United States) was used for plotting and statistical analysis. The Mann–Whitney test was applied for comparisons between two groups, while the Kruskal–Wallis test was used for comparisons among three groups. Spearman’s correlation analysis was performed to assess the potential correlation between the gut microbiota and colitis indices. Values are presented as mean ± SD. *p* ≤ 0.05 was considered statistically significant.

## 3 Results

### 3.1 DSS-induced canine IBD model construction

**Figure 2:**
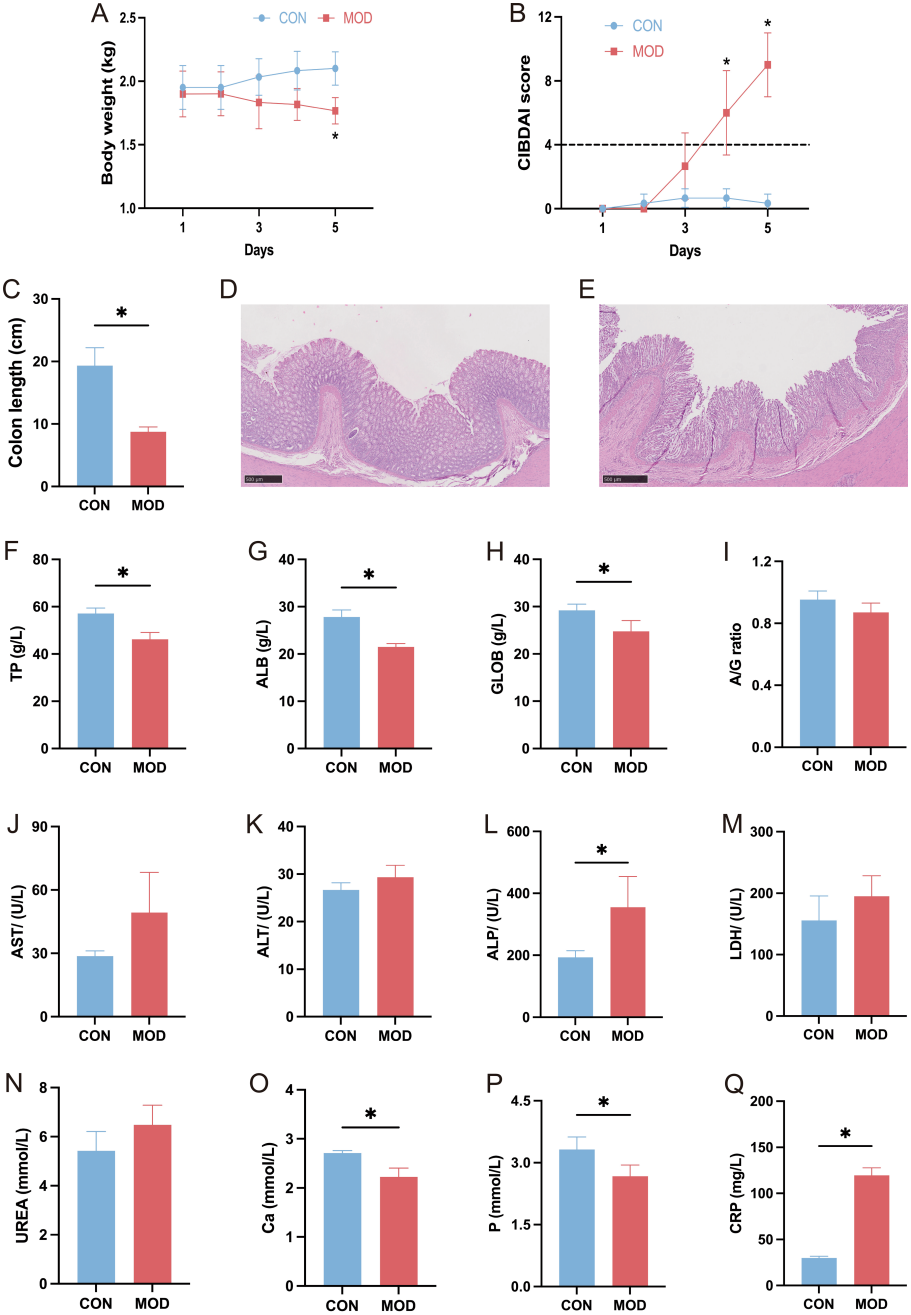
Construction of the DSS-induced canine IBD model. Changes in body weight **(A)**, CIBDAI score **(B)**, and colon length **(C)** in model dogs. HE staining of colonic tissue sections from the CON **(D)** and MOD **(E)** groups. Changes in blood parameters: TP **(F)**, ALB **(G)**, GLOB **(H)**, A/G ratio **(I)**, AST **(J)**, ALT **(K)**, ALP **(L)**, LDH **(M)**, UREA **(N)**, Ca **(O)**, P **(P)**, and CRP **(Q)**. * indicates *p* < 0.05; the same applies hereafter.

The study first evaluated changes in body weight, CIBDAI score, colon length, colonic morphology, and blood parameters in the DSS-induced canine IBD model. As shown in Figure 2A, the body weight of the model dogs gradually declined over the 5-day period, with a significant reduction observed on day 5 (*p* < 0.05). Additionally, the CIBDAI score significantly increased on days 4 and 5 in the model dogs (*p* < 0.05, Figure 2B). Next, colon length was measured, and histological evaluation was performed. Compared to the CON group, the MOD group exhibited a significantly shorter colon length (*p* < 0.05, Figure 2C). Histological analysis using HE staining showed that, in the CON group, the colonic mucosa was intact, epithelial cells were neatly arranged, and no infiltration of inflammatory cells was observed (Figure 2D). In contrast, the MOD group exhibited severe mucosal epithelial damage, abnormal crypt morphology, a reduction in goblet cells, and massive infiltration of inflammatory cells (Figure 2E). Blood parameter results are presented in Figures 2F–Q. Compared to the CON group, TP, ALB, GLOB, Ca, and P levels were significantly decreased, while ALP and CRP levels were significantly increased in the MOD group (*p* < 0.05).

### 3.2 Disrupted gut microbial diversity and composition in DSS-induced IBD dogs

**Figure 3:**
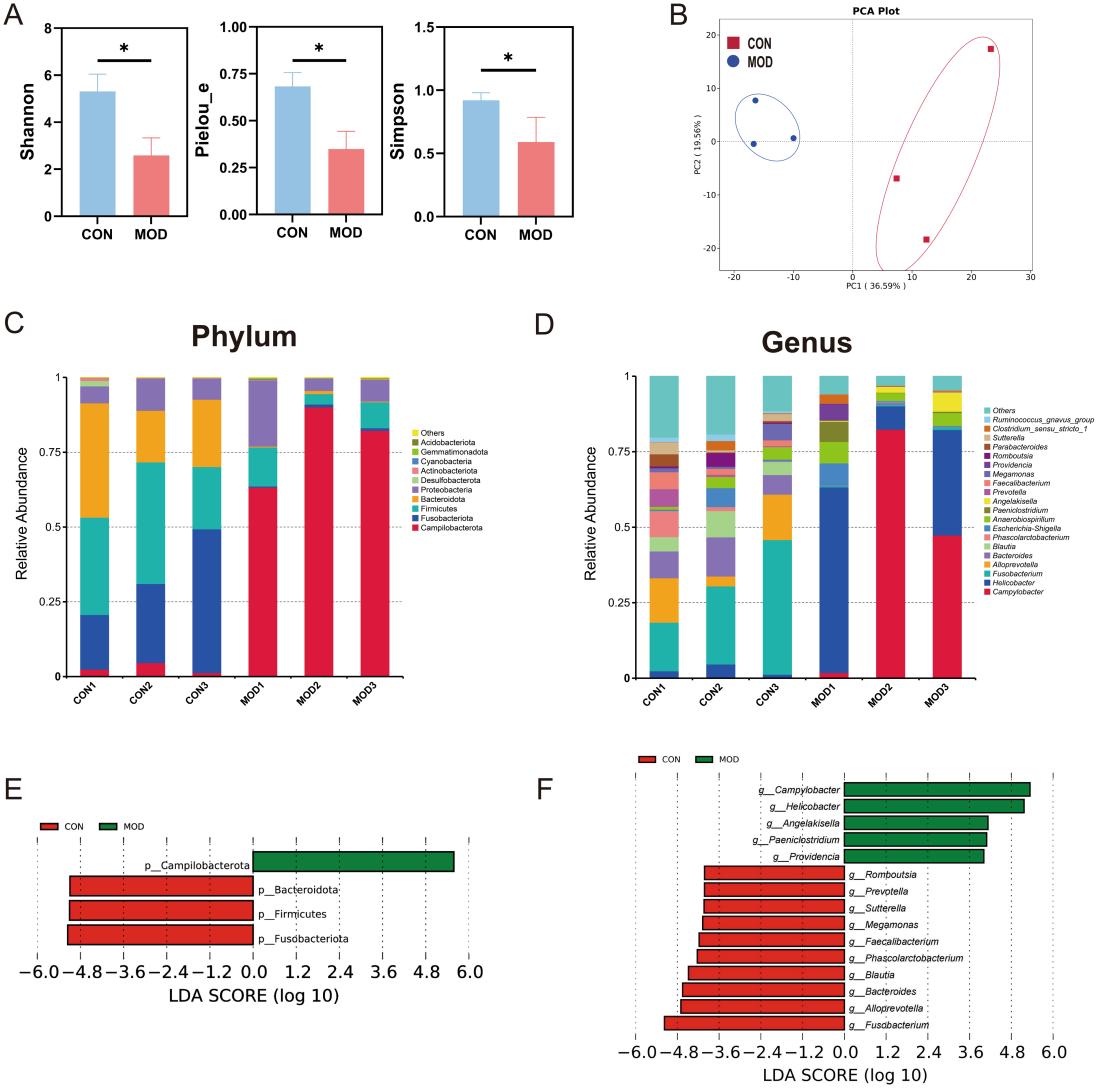
Analysis of intestinal bacterial composition in DSS-induced IBD dogs. **(A)** Comparison of alpha diversity. **(B)** Comparison of beta diversity based on PCA. Relative abundance of bacterial taxa at the phylum **(C)** and genus **(D)** levels. LEfSe histograms showing taxonomic LDA scores higher than 4.0 at the phylum **(E)** and genus **(F)** levels.

To investigate changes in intestinal bacterial communities in DSS-induced IBD dogs, colonic mucosa samples were collected. Using 16S rRNA gene sequencing, a total of 775 amplicon sequencing variants (ASVs) were identified. Rarefaction curves for Observed_otus and Shannon index reached a plateau across all samples, indicating sufficient sequencing depth (Supplementary Figure S1). The analysis first focused on differences in bacterial alpha and beta diversity between two groups. Results showed that the Shannon, Pielou_e, and Simpson indices were significantly reduced in the MOD group, indicating a notable decline in the richness and diversity of intestinal bacteria in DSS-induced IBD dogs (*p* < 0.05, Figure 3A). Furthermore, PCA revealed a clear distinction in the composition of intestinal bacterial communities between two groups (Figure 3B).

Subsequent, differential analysis of intestinal microbiota composition between the CON and MOD groups revealed notable differences. At the phylum level, Campilobacterota, Firmicutes, and Proteobacteria dominated the MOD group, whereas Fusobacteriota, Firmicutes, Bacteroidota, and Proteobacteria were predominant in the CON group (Figure 3C). At the genus level, *Campylobacter* and *Helicobacter* were most abundant in the MOD group, contrasting with *Fusobacterium*, *Alloprevotella*, and *Bacteroides* in the CON group (Figure 3D). To identify differentially abundant bacterial taxa, LEfSe analysis (LDA > 4.0) was conducted. Campilobacterota was identified as a biomarker for the MOD group, while Bacteroidota, Firmicutes, and Fusobacteriota were identified as biomarkers for the CON group (Figure 3E). At the genus level, 15 genera showed differential abundances between the two groups. *Campylobacter*, *Helicobacter*, *Angelakisella*, *Paeniclostridium*, and *Providencia* were identified as biomakers for the MOD group, whereas *Fusobacterium*, Alloprevotella, *Bacteroides*, *Blautia*, *Phascolarctobacterium*, *Faecalibacterium*, *Megamonas*, *Sutterella*, *Prevotella*, and *Romboutsia* were identified as biomarkers for the CON group (Figure 3F).

### 3.3 Ginseng polysaccharides alleviated DSS-induced colitis in dogs

**Figure 4:**
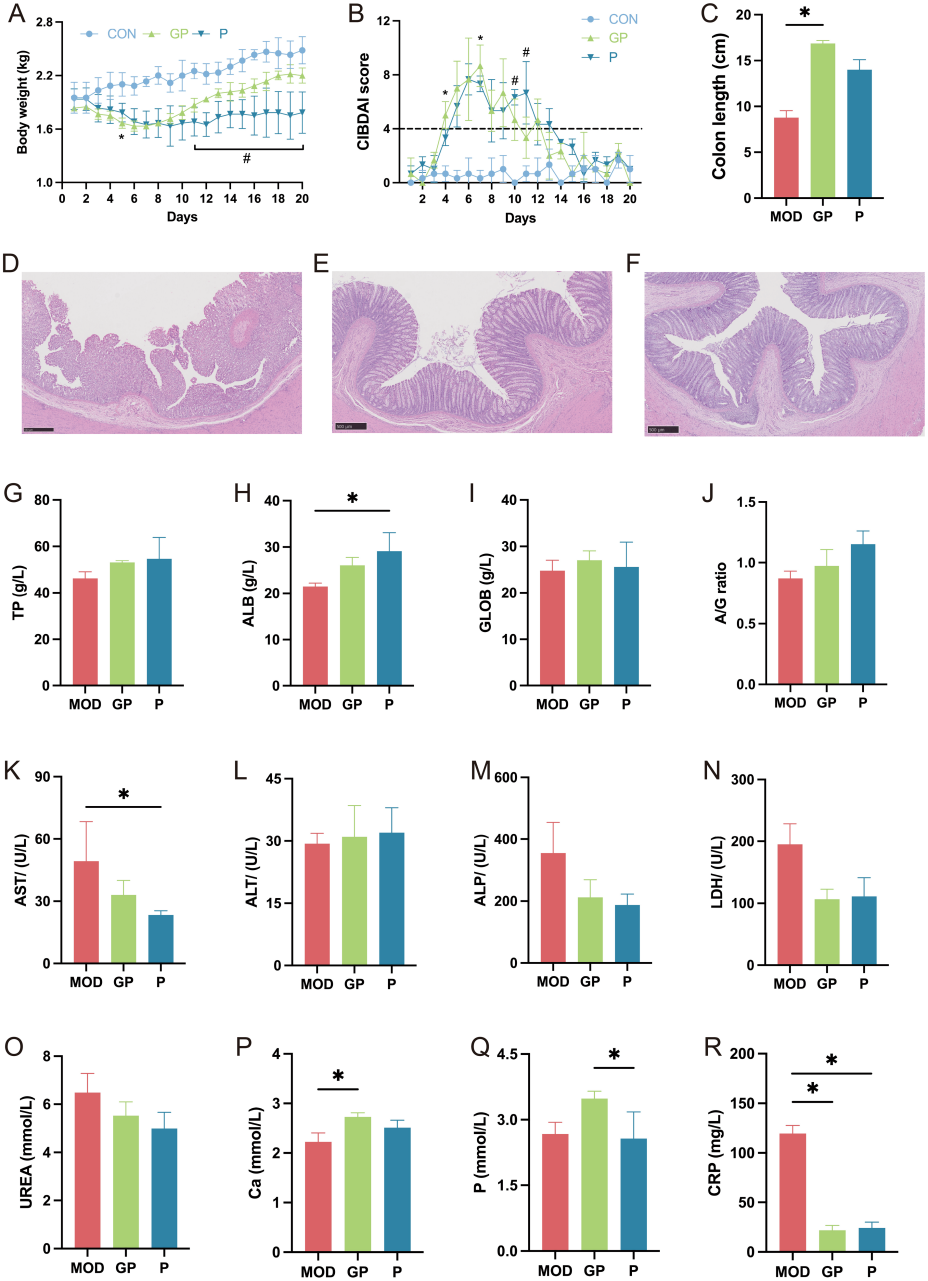
Effects of ginseng polysaccharides on DSS-induced colitis in dogs. Changes in body weight **(A)**, CIBDAI score **(B)**, and colon length **(C)**. Colon tissue histology from the MOD **(D)**, GP **(E)**, and P **(F)** groups. Changes in blood parameter levels: TP **(G)**, ALB **(H)**, GLOB **(I)**, A/G ratio **(J)**, AST **(K)**, ALT **(L)**, ALP **(M)**, LDH **(N)**, UREA **(O)**, Ca **(P)**, P **(Q)**, and CRP **(R)**. In **(A,B)**, * indicates comparisons between the GP and CON groups, and # indicates comparisons between the P and CON groups.

To evaluate the therapeutic effects of ginseng polysaccharides on DSS-induced IBD in dogs, changes in body weight, CIBDAI score, colon length, colon tissue histology, and serum parameters were assessed. As shown in Figure 4A, body weight in the GP group significantly decreased on day 5 compared to the CON group (*p* < 0.05). In contrast, the P group exhibited a significant weight reduction from day 11 to day 20 (*p* < 0.05). Moreover, the GP group showed weight gain, whereas the P group displayed a decline by the end of the experiment relative to their respective initial weights. The CIBDAI scores in both the GP and P groups showed a similar downward trend (Figure 4B). Regarding colon length, the GP group displayed a significant increase compared to the MOD group (*p* < 0.05, Figure 4C). Histopathological analysis of the colon revealed mucosal erosion, crypt damage, goblet cell loss, and inflammatory cell infiltration in the MOD group (Figure 4D). In contrast, treatment with ginseng polysaccharides and prednisone markedly alleviated histological damage, with the mucosal barrier remaining nearly intact and a clear reduction in epithelial inflammatory cell infiltration (Figures 4E,F). Serum parameter analysis showed that TP, ALB, GLOB, P levels, and the A/G ratio were slightly higher in the GP group than in the MOD group, although the differences were not statistically significant (*p* > 0.05; Figures 4G–J,Q). No changes in ALT activities were observed among the three groups (Figure 4L). AST, ALP, LDH, and UREA levels were slightly lower in the GP group, but these differences were also not significant (*p* > 0.05; Figures 4K,M–O). Notably, Ca levels were significantly increased, and CRP levels significantly decreased in the GP group relative to the MOD group (*p* < 0.05; Figures 4P,R).

### 3.4 Ginseng polysaccharides improved DSS-induced gut microbiota disorder in dogs

**Figure 5:**
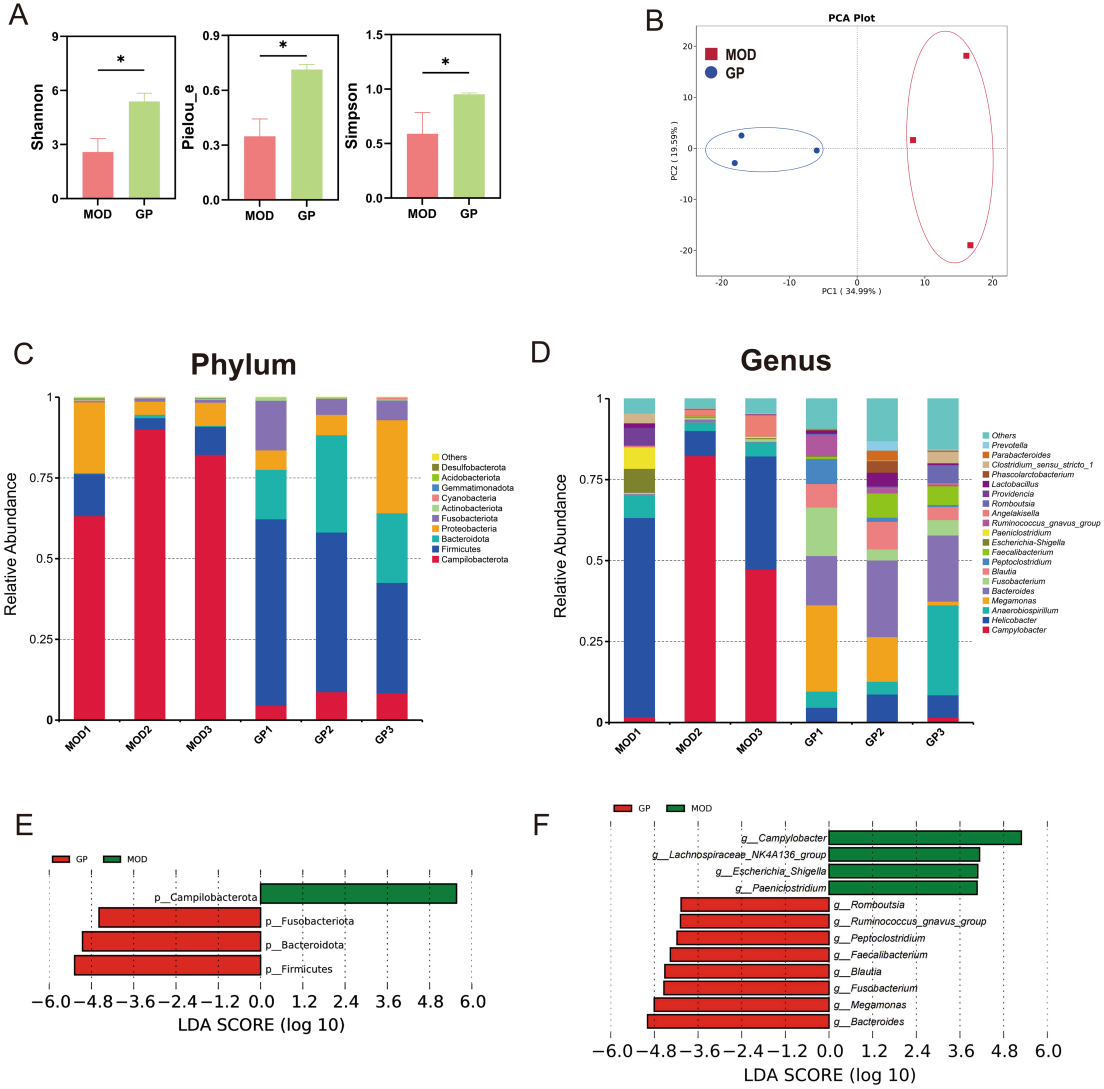
Effects of ginseng polysaccharides on the gut microbiota in DSS-induced IBD dogs. Comparison of alpha diversity **(A)** and beta diversity **(B)** between the MOD and GP groups. Relative abundances at the phylum **(C)** and genus **(D)** levels. LEfSe histograms at the phylum **(E)** and genus **(F)** levels.

The study further examined the effect of ginseng polysaccharide treatment on the gut microbiota in DSS-induced IBD dogs. Results showed that ginseng polysaccharide treatment significantly increased the alpha diversity of the gut microbiota, indicating higher community richness and bacterial diversity in the GP group compared to the MOD group (*p* < 0.05, Figure 5A). PCA revealed a distinct microbial community structure between the GP and MOD groups (Figure 5B). At the phylum level, the gut microbiota of the MOD group was predominantly composed of Campilobacterota, Proteobacteria, and Firmicutes, whereas the GP group was dominated by Firmicutes, Bacteroidota, Proteobacteria, and Fusobacteriota (Figure 5C). At the genus level, the results showed that the MOD group was dominated by *Campylobacter* and *Helicobacter*, while the GP group was dominated by *Anaerobiospirillum*, *Megamonas*, and *Bacteroides* (Figure 5D). LEfSe analysis (LDA > 4.0) identified Campilobacterota as a biomarker for the MOD group, while Firmicutes, Bacteroidota, and Fusobacteriota were identified as biomarkers for the GP group (Figure 5E). At the genus level, *Campylobacter*, *Lachnospiraceae_NK4A136_group*, *Escherichia_Shigella*, and *Paeniclostridium* were identified as biomarkers for the MOD group (Figure 5F). In contrast, *Bacteroides*, *Megamonas*, *Fusobacterium*, *Blautia*, *Faecalibacterium*, *Peptoclostridium*, *Ruminococcus_gnavus_group*, and *Romboutsia* were identified as biomarkers for the GP group (Figure 5F).

### 3.5 Ginseng polysaccharides enhanced intestinal SCFAs production in DSS-induced IBD dogs

**Figure 6:**
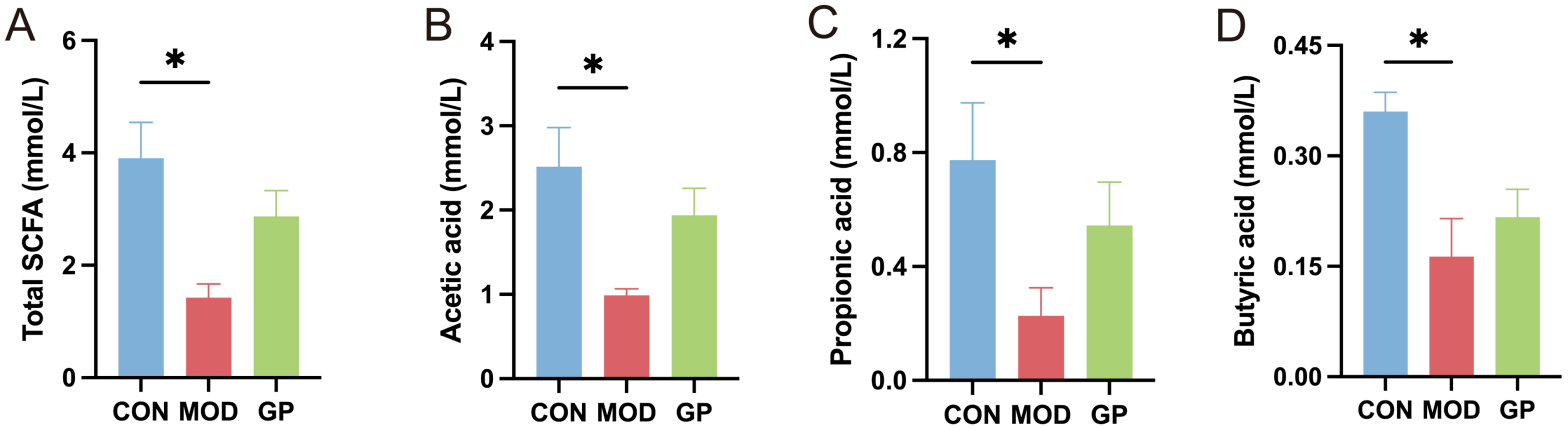
Effects of ginseng polysaccharides on SCFA production in DSS-induced IBD dogs. Comparison of the levels of total SCFA **(A)**, acetic acid **(B)**, propionic acid **(C)**, and butyric acid **(D)** among the CON, MOD, and GP groups.

SCFAs, important metabolites produced by intestinal bacteria, were also evaluated in this study. As shown in Figure 6, the MOD group exhibited a significant reduction in the levels of total SCFAs, acetic acid, propionic acid, and butyric acid compared to the CON group (*p* < 0.05). Treatment with ginseng polysaccharides promoted the production of total SCFAs, acetic acid, propionic acid, and butyric acid in IBD dogs, although these increases were not statistically significant (*p* > 0.05).

### 3.6 Prediction of gut microbiota biological functions

**Figure 7:**
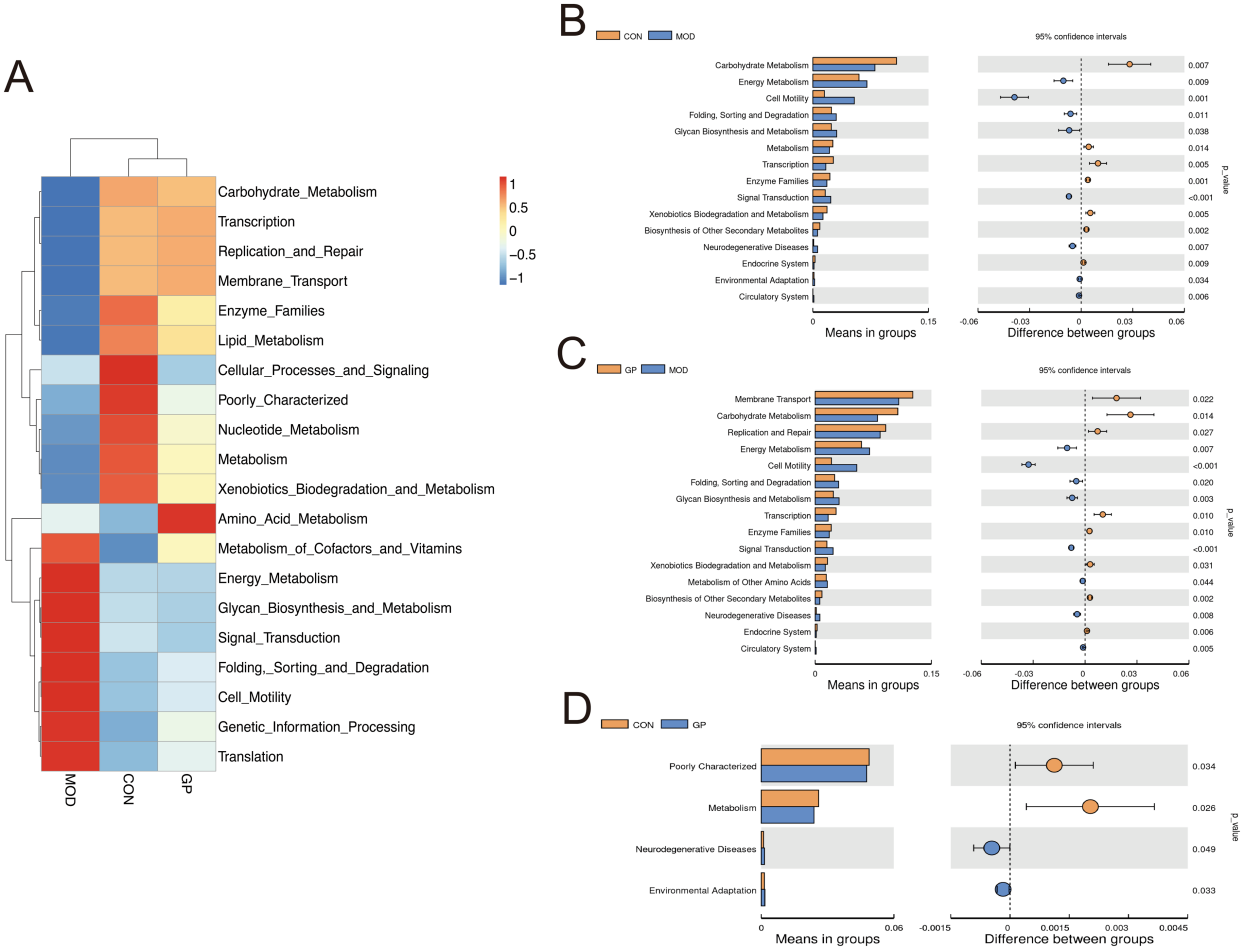
Predicted functional changes in the gut microbiota based on PICRUSt analysis. **(A)** Heatmap showing the top 20 most enriched KEGG pathways (Level 2). Red indicates unregulated pathways, while blue indicates downregulated pathways. **(B–D)** Differentially enriched pathways based on pairwise comparisons among the three groups.

The PICRUSt analysis was conducted to predict the functional profiles of the gut microbiota across the three groups. A heatmap displaying the top 20 most enriched microbial pathways at Level 2 is shown in Figure 7A. Compared with the CON and GP groups, DSS treatment upregulated pathways related to energy metabolism, glycan biosynthesis and metabolism, signal transduction, folding, sorting and degradation, cell motility, genetic information processing, and translation. In contrast, pathways involved in carbohydrate metabolism, transcription, replication and repair, membrane transport, enzyme families, and lipid metabolism were downregulated.

Further *t*-test analysis revealed similar results: compared with the CON group, the MOD group showed significant upregulation in pathways associated with energy metabolism, cell motility, folding, sorting and degradation, glycan biosynthesis and metabolism, and signal transduction (*p* < 0.05; Figure 7B). At the same time, it showed significant downregulation in pathways related to carbohydrate metabolism, metabolism, transcription, enzyme families, xenobiotics biodegradation and metabolism, and biosynthesis of other secondary metabolites (*p* < 0.05; Figure 7B). Importantly, GP treatment significantly reversed these DSS-induced functional changes in the gut microbiota (Figure 7C). The overall functional profile of the GP group closely resembled that of the CON group (Figure 7D).

### 3.7 Correlation analysis between colitis indices and gut microbiota

**Figure 8:**
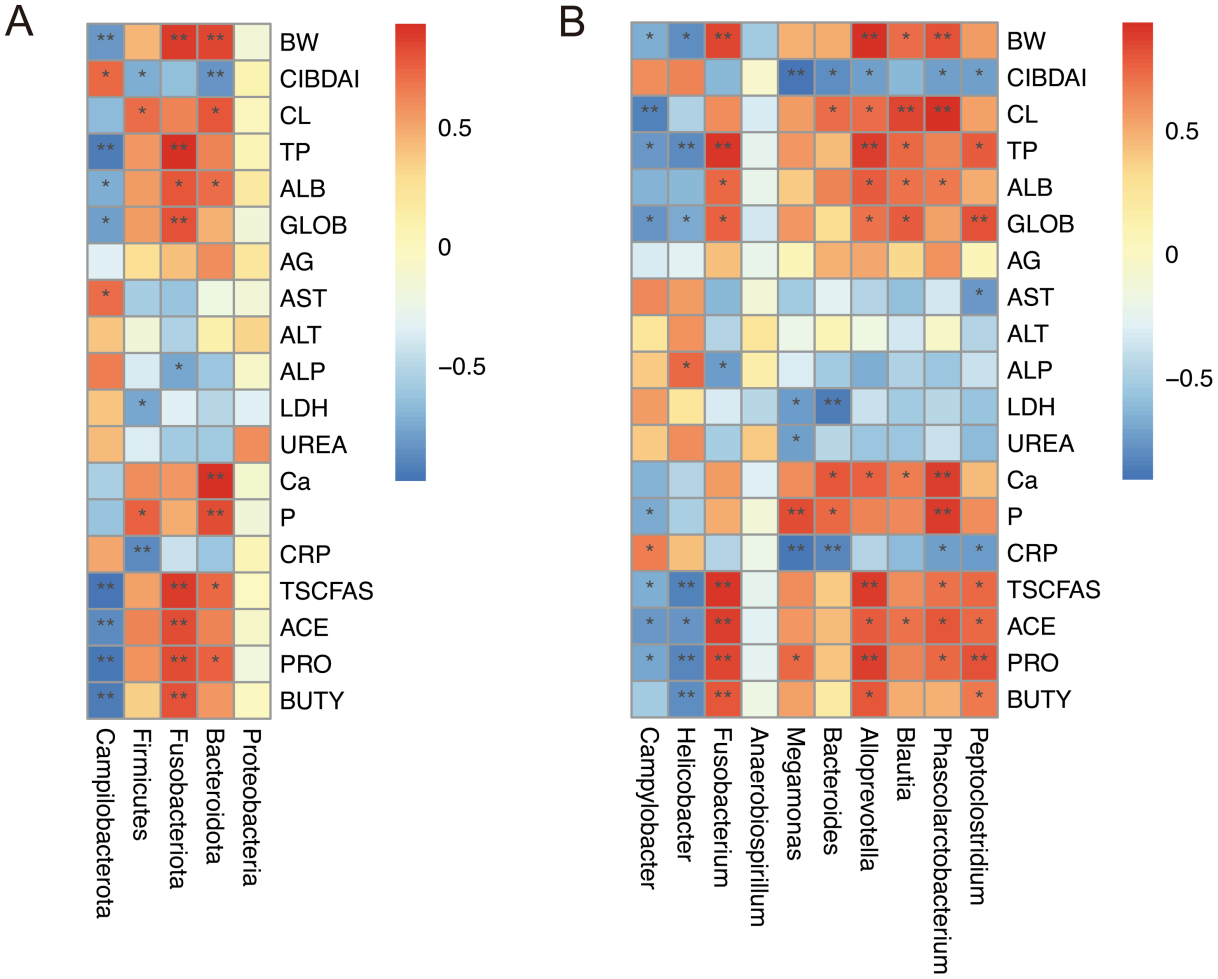
Spearman’s correlation analysis between colitis indices and gut microbiota at the phylum **(A)** and genus **(B)** levels. BW, body weight; CL, colon length; AG, A/G ratio; TSCFAS, total SCFAs; ACE, acetic acid; PRO, propionic acid; BUTY, butyric acid. Red represents a positive correlation, while blue represents a negative correlation. * indicates *p* < 0.05, ** indicates *p* < 0.01.

To comprehensively evaluate the correlation between colitis indicators and gut microbiota composition, Spearman’s correlation analysis was performed. As shown in Figure 8A, at the phylum level, Campilobacterota abundance was positively correlated with the CIBDAI score and AST activity, but negatively correlated with body weight, TP levels, and total SCFAs. Firmicutes were positively associated with colon length and P levels, and negatively associated with the CIBDAI score, LDH activity, and CRP levels. Fusobacteriota showed positive correlations with body weight, TP levels, and total SCFAs, and a negative correlation with ALP activity. Bacteroidota were positively correlated with body weight, colon length, ALB, Ca and P levels, and total SCFAs, while negatively correlated with the CIBDAI score.

At the genus level (Figure 8B), *Campylobacter* abundance was positively correlated with CRP levels, and negatively correlated with body weight, colon length, TP and P levels, and total SCFAs. *Helicobacter* was positively correlated with ALP activity and negatively correlated with body weight, TP levels, and total SCFAs. In contrast, *Fusobacterium*, *Alloprevotella*, and *Phascolarctobacterium* abundances were positively correlated with body weight, colon length, TP levels, Ca and P levels, and total SCFAs, and negatively correlated with the CIBDAI score and CRP levels.

## 4 Discussion

The pathogenesis of IBD remains unclear, although it is widely accepted to involve environmental, hereditary, immune, and gut microbiota-related factors (33). Currently, there is no standardized therapy, and the overall prognosis is generally unfavorable. Because anti-inflammatory drugs show limited efficacy and may cause notable side effects, plant-derived natural compounds have received increasing attention as potential alternatives for IBD prevention and treatment (34). Among experimental models, the DSS-induced colitis model is widely used in IBD research to investigate disease mechanisms and explore therapeutic strategies.

In this study, we established a DSS-induced colitis model in dogs based on our previous work (30). The model dogs exhibited clinical features similar to those observed in mouse and rat models, including weight loss, elevated CIBDAI scores, shortened colon length, damaged colon tissue, and colon inflammation (35–38). Notably, treatment with ginseng polysaccharides significantly ameliorated colitis symptoms, showing effects comparable to those of prednisone, including reduced CIBDAI scores, restoration of colon length, and decreased pathological damage to colon tissue. A previous study found that ginseng polysaccharides exert immunomodulatory effects by promoting the proliferation of immune cells such as natural killer cells, lymphocytes, and macrophages (39). A recent study also demonstrated that ginseng polysaccharide treatment significantly improved symptoms of DSS-induced ulcerative colitis in mice by enhancing the activity of antioxidant enzymes (SOD and CAT), reducing MDA levels, and suppressing pro-inflammatory cytokines such as TNF-α, IL-1β, and IL-6 (40). Based on these findings, we speculate that the therapeutic effects of ginseng polysaccharides in colitis dogs may be related to enhanced antioxidant activity, reduced inflammation, and improved immune function. Additionally, dogs treated with ginseng polysaccharides gained more weight by the end of the study than those treated with prednisone. This may be due to ginseng polysaccharides enhancing food intake by regulating circulating glucose levels (41) and promoting digestion and nutrient absorption by restoring intestinal microbial balance (42, 43).

Although IBD primarily affects the gastrointestinal tract, it is a systemic disease that may involve multiple organs, including the skin, bones, heart, liver, and kidneys, leading to a broad range of extraintestinal manifestations (44, 45). These complications are thought to arise from immune dysfunction, release of inflammatory mediators, impaired nutrient absorption, gut microbiota imbalance, and genetic susceptibility (46). Serum biochemical parameters are valuable indicators of an animal’s physiological and metabolic status and provide insight into overall health (47). In this study, we assessed changes in these parameters among different groups. Compared with the CON group, the MOD group showed significantly decreased levels of TP, ALB, and GLOB, along with a modest reduction in the A/G ratio. Serum Ca and P levels were also markedly reduced. Serum TP, composed of ALB and GLOB, serves as an important indicator of inflammation and nutritional status (48), while Ca and P are commonly used as indicators of vitamin D deficiency (49). In IBD, persistent diarrhea and vomiting often lead to reduced food intake, and when combined with gut dysbiosis and mucosal inflammation, digestion and nutrient absorption are further impaired (50, 51). These factors collectively contribute to malnutrition, which can manifest as declines in multiple serum biochemical parameters. Our results showed that these serum parameters improved to varying degrees after treatment with ginseng polysaccharides, although only the change in Ca levels reached statistical significance. The overall improvement may be related to enhanced appetite, accelerated repair of the intestinal mucosa, and restoration of gut microbiota balance (52, 53), which together contribute to better nutritional status.

Serum AST, ALT, ALP, and LDH are commonly used indicators for assessing liver injury (54). UREA, the primary end product of protein metabolism, is synthesized in the liver and excreted by the kidneys (55), making it a useful biomarker for dehydration and an important parameter for evaluating kidney function (56). CRP is an acute-phase protein produced by the liver and serves as a non-specific marker of inflammation, with serum levels increasing during inflammatory responses (57). In this study, we measured AST, ALT, ALP, LDH, UREA, and CRP across all groups. In IBD dogs, these parameters generally showed upward trends, which may be related to systemic inflammation and dehydration caused by persistent diarrhea. However, given the small sample size in each group (*n* = 3), most changes did not reach statistical significance. After treatment with ginseng polysaccharides, several indicators, particularly AST, ALP, LDH, and UREA, showed downward trends, although again without statistical significance. Only CRP exhibited a clear and significant reduction. These non-significant trends should be interpreted with caution, as the limited sample size may not provide sufficient statistical power to detect true inter-group differences. Even so, the observed tendencies are consistent with the known anti-inflammatory properties of ginseng polysaccharides (58) and their reported ability to improve intestinal barrier function (59), which may help alleviate clinical symptoms. Future studies with larger sample sizes are needed to confirm these preliminary observations.

Alterations in the intestinal microbiota are recognized as an important factor in IBD. Although a definitive causal relationship has yet to be established, both the microbiota and its metabolites play critical roles in disease progression (60). Consistent with previous reports, dogs with IBD in this study showed a significant reduction in microbial richness and diversity, along with disruption of the overall microbial community structure (61). Notably, treatment with ginseng polysaccharides effectively mitigated these imbalances. In the GP group, alpha diversity (Shannon, Pielou_e, and Simpson indices) was significantly increased, indicating improved microbial stability. Moreover, ginseng polysaccharide treatment altered microbial composition. At the phylum level, there was a significant increase in the relative abundance of Firmicutes, Bacteroidota, and Fusobacteriota, accompanied by a notable reduction in Campilobacterota. Firmicutes, Bacteroidota, and Fusobacteriota are predominant phyla in the canine gut and are crucial for maintaining intestinal homeostasis and overall health (62). Firmicutes and Bacteroidota encode numerous enzymes responsible for the degradation of complex carbohydrates (63). Firmicutes mainly produce butyrate, while Bacteroidota generate acetate and propionate as their primary end-products (64). Fusobacteriota also contribute significantly to butyrate production (65). These SCFAs not only serve as energy sources but also possess anti-inflammatory properties and help maintain gut equilibrium (66, 67). In contrast, Campilobacterota is a pathogenic phylum commonly associated with gastrointestinal disorders and autoimmune diseases (68).

At the genus level, ginseng polysaccharide treatment significantly reduced the relative abundance of pathogenic genera, including *Campylobacter*, *Escherichia-Shigella*, and *Paeniclostridium*. A decrease in *Helicobacter* was also observed, though it did not reach statistical significance. These genera are closely associated with intestinal inflammation (69–71). In contrast, the treatment significantly increased the relative abundance of *Bacteroides*, *Megamonas*, *Fusobacterium*, *Blautia*, *Faecalibacterium*, *Peptoclostridium*, *Ruminococcus_gnavus_group*, and *Romboutsia*, genera commonly present in the healthy canine gut (72, 73). The enrichment of these beneficial microbes suggests a restoration of gut microbial balance.

Our findings showed that total SCFAs, including acetate, propionate, and butyrate, were markedly reduced in dogs with IBD. After treatment with ginseng polysaccharides, the levels of these fatty acids showed an upward trend, although none of the changes reached statistical significance. Given the small sample size in each group (*n* = 3), these results should be interpreted cautiously as trends rather than definitive effects. PICRUSt analysis indicated that carbohydrate metabolism was impaired in dogs with IBD, while ginseng polysaccharide treatment significantly improved this metabolic function. Correspondingly, shifts in gut microbiota composition were observed in several bacterial taxa associated with carbohydrate utilization. Overall, the microbiota changes, metabolic predictions, and SCFA trends provide preliminary insight into the potential effects of ginseng polysaccharides in DSS-induced canine IBD.

In addition, Spearman correlation analysis showed that reductions in pathogenic bacteria, coupled with increases in beneficial microbes, were strongly associated with improvements in clinical symptoms and serum parameters. These associations highlight a close relationship between microbiota composition and the progression of canine IBD. The observed microbial shifts may reflect the prebiotic properties of ginseng polysaccharides, which selectively stimulate the growth and activity of beneficial bacteria while inhibiting pathogenic species, thereby supporting the restoration of gut microecological homeostasis (15, 16).

Despite the promising findings, several limitations warrant consideration. Foremost, each experimental group contained only three dogs, a constraint driven mostly by ethical considerations. This small sample size reduces statistical power, particularly for microbiome analyses and SCFA measurements, and may limit the generalizability of the results. Accordingly, several observations in this study should be interpreted as trends rather than definitive effects. Additionally, although the study provides extensive clinical, biochemical, histological, and microbial data, mechanistic insights at the molecular level were not explored. Future studies integrating transcriptomic, proteomic, or metabolomic analyses would help elucidate the signaling pathways and molecular interactions underlying the therapeutic effects of ginseng polysaccharides. Finally, the use of an acute DSS-induced model may not fully replicate the chronic and heterogeneous nature of spontaneous canine IBD; therefore, clinical trials in naturally affected dogs would further support the translational relevance of our findings. Overall, this study offers foundational evidence that ginseng polysaccharides may alleviate DSS-induced colitis in dogs through improvements in clinical symptoms, serum biochemical profiles, and gut microbial balance. While these preliminary findings are encouraging, further studies with larger cohorts and greater mechanistic depth are needed to substantiate the therapeutic potential of ginseng polysaccharides for canine IBD.

## 5 Conclusion

In conclusion, this study shows that ginseng polysaccharides mitigate DSS-induced IBD in dogs, improving clinical signs, serum biochemical changes, histopathological injury, and gut microbiota disturbances. Although most findings were not statistically significant due to the small sample size, the observed trends suggest potential therapeutic value. These results provide preliminary evidence supporting ginseng polysaccharides as a candidate for canine IBD treatment.

## Data Availability

The datasets presented in this study can be found in online repositories. The names of the repository/repositories and accession number(s) can be found at: https://www.ncbi.nlm.nih.gov/, PRJNA1332772.
