# Supplementary figures and images for: Ginseng polysaccharides ameliorate DSS-induced inflammatory bowel disease by regulating gut microbiota in dogs
Source: Front Vet Sci. 2026 Jan 8;12:1708594. doi: 10.3389/fvets.2025.1708594 (PMC12823840; doi:10.3389/fvets.2025.1708594)

## Supplementary Material

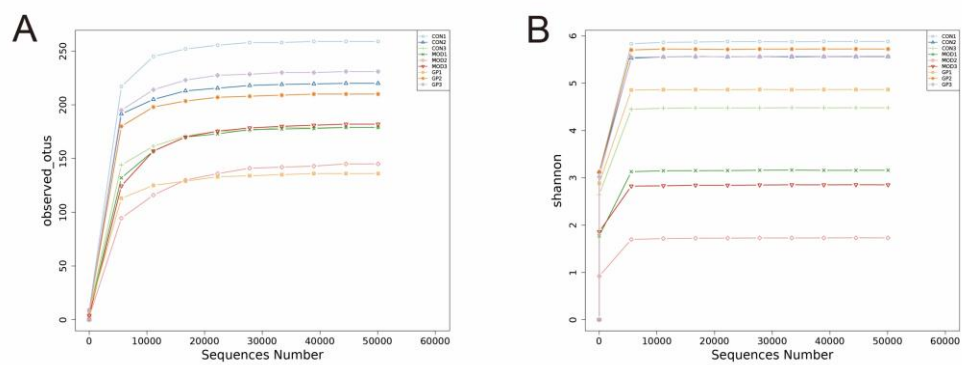

Figure S1. Rarefaction curves of Observed\_otus (A) and Shannon index (B).

Supplement: Supplementary file 1 [file Image_1.pdf]
